# Copper Nanowires and Their Applications for Flexible, Transparent Conducting Films: A Review

**DOI:** 10.3390/nano6030047

**Published:** 2016-03-09

**Authors:** Vu Binh Nam, Daeho Lee

**Affiliations:** Department of Mechanical Engineering, Gachon University, Seongnam 13120, Korea; vubinhnam@gc.gachon.ac.kr

**Keywords:** copper nanowires, optoelectronic properties, transparent conducting films, flexible, protection, ITO replacement

## Abstract

Cu nanowires (NWs) are attracting considerable attention as alternatives to Ag NWs for next-generation transparent conductors, replacing indium tin oxide (ITO) and micro metal grids. Cu NWs hold great promise for low-cost fabrication via a solution-processed route and show preponderant optical, electrical, and mechanical properties. In this study, we report a summary of recent advances in research on Cu NWs, covering the optoelectronic properties, synthesis routes, deposition methods to fabricate flexible transparent conducting films, and their potential applications. This review also examines the approaches on protecting Cu NWs from oxidation in air environments.

## 1. Introduction

The demand for transparent screens is huge and increasing rapidly. From large flat-panel displays to small smartphones and smart watches, the devices we use and encounter every day and everywhere need transparent conducting films (TCFs). Also, optoelectronic devices such as thin-film solar cells [1,2], organic light-emitting diodes (OLEDs) [3,4], thermo-electrics [5], and sensors [6] utilize TCFs as their substrates. Moreover, flexibility is becoming an additional requirement for TCFs as the demand for flexible electronics is growing rapidly. In the current transparent conductor industry, indium tin oxide (ITO) films coated on glass occupy a majority of the market. However, current ITO films are deposited using a vacuum process, which requires expensive equipment and generates significant material waste. Furthermore, ITO films cannot withstand repeated bending or rolling because they are brittle and prone to cracking, limiting their use in truly flexible devices [7]. More importantly, indium, which is a rare material, causes fluctuations in ITO prices [8]. As a result, the use of traditional ITO-based TCFs led to delays in terms of new production and innovations. The developed markets for flexible and curved screens require transparent conductor materials that can be processed at low cost on plastic substrates and that have excellent mechanical and optoelectronic performance. Thus, many researchers have investigated alternative materials for ITO for use in flexible transparent conducting electrodes, such as carbon-based nanomaterials (nanotubes [9,10] and graphene [11,12]), zinc oxide (ZnO)-based transparent conducting oxides [13,14,15], noble metals (Au, Ag) [16,17], metal micro grids [18,19], nanowire grids [20,21], and graphene–silver nanowire (NW) hybrids [22]. However, conducting networks of carbon-based nanomaterials, including carbon nanotubes (CNTs) and graphene, exhibit relatively poor optoelectronic performance as compared to ITO [23,24]. The high price of noble metals makes these metals uncompetitive for large-area, low-cost transparent conductors, even though they exhibit superior conductivity and transmittance. Recently, noble metal NWs have emerged as promising materials for achieving performance equivalent to that of ITO. Ag NWs achieved a transparency with a low sheet resistance (*R*_s_), which surpasses the ITO performance [25]. Ag NWs also can be deposited on flexible substrates by spin coating or vacuum transfer [18,26,27]. However, the high material cost of Ag still presents a challenge to the mass production of optoelectronic devices.

Copper (Cu) NWs have recently garnered increasing attention as excellent candidates for TCFs. Because Cu has high intrinsic conductivity, it is a potential alternative to ITO and Ag NWs. Moreover, Cu is only 6% less conductive than Ag, very abundant (1000 times more abundant than Ag), and 100 times less expensive than Ag or ITO [28]. Several studies have been reported, demonstrating how Cu NW films can achieve high optical transmittance with high electric conductivity [29,30,31]. However, challenges remain for the stabilization of Cu NWs because they are prone to oxidation under ambient conditions, and thus they cannot retain performance comparable to that of ITO.

In this review, we report recent research progress on Cu NWs as promising transparent conducting materials. This review covers the optoelectronic properties, synthesis routes of Cu NWs, deposition methods to fabricate TCFs, and their potential applications. Firstly, we summarize the relationship between the physical dimensions and optoelectronic properties of metal NWs. In the second part, the synthesis routes of Cu NWs and the fabrication processes of Cu NW thin films are reviewed. In the third part, we discuss how to protect and passivate Cu NWs from oxidation. Finally, the applications of Cu NWs and required future studies are proposed.

## 2. Relationship of Optoelectronic Properties and Nanowire Dimensions

### 2.1. Electrical Properties of Nanowire Films

High-quality TCFs require both high conductivity and high transmittance [32]. In general, however, a metal thin film with high conductivity leads to low transmittance because the conductivity is proportional to the film thickness [33]. A functional relationship between NW dimensions and the properties of NW-based films is very important and useful for the synthesis of NWs optimized for use in transparent electrodes. In this part, we summarize the structure–property correlations of NW films based on recently published articles. Although many theories and experiments introduced here have been established and performed with materials other than Cu NWs, we can predict the properties of Cu NWs based on these previous studies.

**Figure 1:**
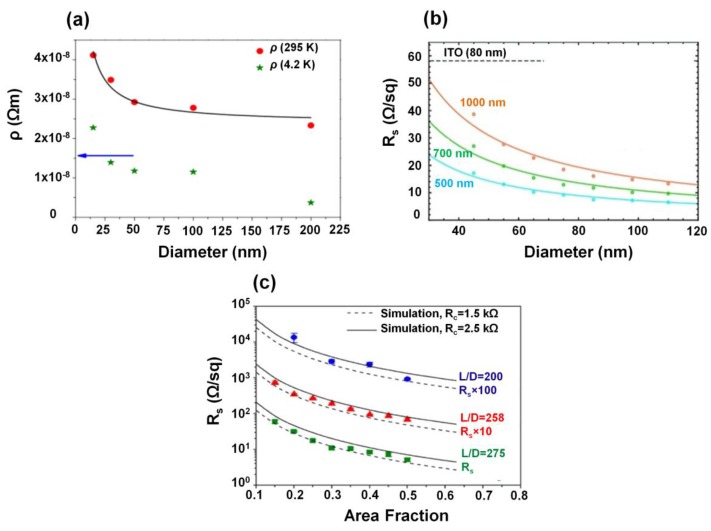
(**a**) Plot of the resistivity (*ρ*) of Ag nanowires (NWs) at 295 K and 4.2 K and the residual resistivity as a function of the NW diameter. The line is the fit to the data using Equation (1). The arrow is the bulk value of the resistivity of Ag, 1.629 × 10^−^^8^ Ωm. (Reproduced with permission from [36]. Copyright American Physical Society, 2006); (**b**) The dots represent measured sheet resistances (*R*_s_) as a function of NW width for network pitches of 500 (blue), 700 (green), and 1000 nm (orange). The solid lines represent fitted plots for each pitch. (Reproduced with permission from [38]. Copyright American Chemical Society, 2012); (**c**) Experimental *R*_s_ values for silver NW thin films (points) as a function of the area fraction (AF) for the L/D values indicated. *R*_s_ from quasi-2D simulations of rods with specified L and D values use an effective contact resistance (*R*_c_) to fit the simulations to the experimental data; the best fits correspond to *R*_c_ = 1.5 kΩ (dashed lines) and 2.5 kΩ (solid lines) (Reproduced with permission from [42]. Copyright American Chemical Society, 2013).

The first theory for predicting the dependence of resistivity on thin-film dimensions was introduced by Fuchs *et al.* [34]. Dingle subsequently extended Fuchs’s theory to metal NWs [35]. In this model, the dependence of the resistivity on the diameter of NWs can be expressed as:

(1) $$\rho_{d}=\frac{\rho_{o}+\rho_{o}\left(1-\rho \right)l_{o}}{D}$$

where $\rho_{d}$ is the resistivity of a NW of diameter *D*, $\rho_{o}$ is the bulk or the so-called “intrinsic resistivity” of the NW material, ρ is the specularity factor which denotes the fraction of conduction electrons undergoing specular reflection at the NW surface, and *l_o_* is the free path of the conduction electrons. Figure 1a shows the resistivity dependence of Ag NWs on the diameter [36]. The resistivity decreases as the NW diameter increases, which is attributed to the surface scattering of conduction electrons [33,36,37]. Figure 1b shows the measured *R*_s_ (dots) as a function of the diameter of Ag NW networks with network pitches of 500 (blue), 700 (green), and 1,000 nm (orange) [38]. The solid lines represent the fitted plots of *R*_s_, which confirm that *R*_s_ decreases for all networks as the diameter of the NW increases. Figure 1c shows experimental (points) and simulative (lines) *R*_s_ values for Ag NW thin films as a function of area fraction (AF) for the three L/D values indicated, where AF is defined as the number density of the nanowires in a film multiplied by the projected area of a nanowire. We can verify that the *R*_s_ decreases with increasing AF and L/D.

The effect of NW dimensions on the properties of NW-based TCFs can also be understood by the percolation threshold. Li *et al.* [39] used Monte Carlo simulation to verify that the length of the widthless sticks, *L,* and the critical number density of sticks, *N_c_*, achieves percolation conductivity by the following equation:

*N_c_L*^2^*≈* 5.71 (2)

where 5.71 is the simulation result from extraction of a high-precision *N_c_* value. This equation indicates that double-length NWs require only a quarter of the number density of NWs to achieve percolation. Therefore, if more NWs are added to a film that has just achieved percolation, more conducting pathways formed between NWs increase network conductivity [40].

Recently, Borchert *et al.* integrated simulations and experiments to predict the relationship between *R*_s_ and the dimensions of Cu NWs [41]. They reported that the *R*_s_ value of the Cu NW films decreases as AF increases. In their study, they assumed that all NWs can be modeled as cylinders with a constant diameter, which is taken to be the experimentally obtained average diameter. The AF of a film covered by NWs was calculated by the following equation [41]:

(3) $$AF=\frac{4{\left(Nt\right)}_{i}^{\text{NW}}M_{i}}{\pi \rho_{B}D}$$

where ${\left(Nt\right)}_{i}^{\text{NW}}$ is the atomic areal density of element *i* in the NW sample measured by Rutherford backscattering spectrometry, $M_{i}$ is the molecular weight of the NW component element, $\rho_{B}$ is the bulk atomic density of the material, and *D* is the diameter of the NW.

### 2.2. Optical Properties of Nanowire Films

Controlling optical properties of NW thin films is also critical for TCF applications. Although many studies regarding optical properties of NW films have been performed using materials other than Cu NWs, the fundamental relationship between the NW structure and the optical properties can be applied to general NW films, including Cu NW films. The optical properties of NW films can be determined by considering the surface AF of the NW films. The amount of light blocked by a NW network is equivalent to the light blocked by the AF of the film covered by the NWs. Bergin *et al.* predicted how transmittance is affected by the dimensions of NWs of a random NW network [40]. The transmittance of the film is calculated by

**Figure 2:**
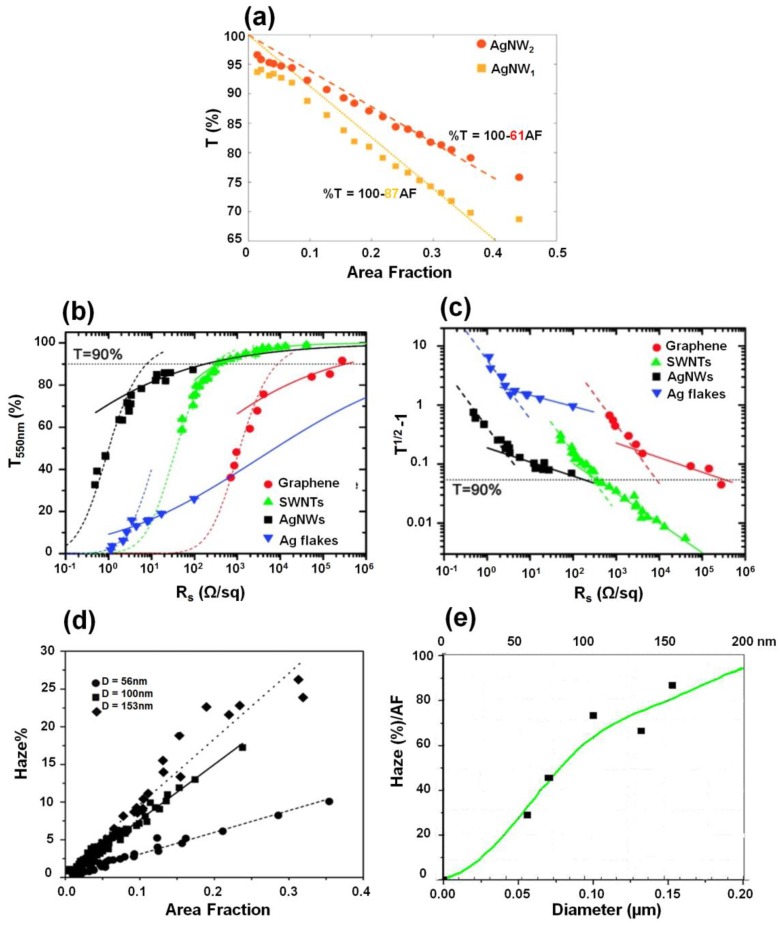
(**a**) The relationship between AF and transmittance (*T*). Different L/D in AgNW1 and AgNW2 lead to different trends in both *T* and *R_s_*. (Reproduced with permission from [43]. Copyright Springer Science + Business Media on behalf of the American Coatings Association and the Oil and Colour Chemist Association, 2015); (**b**) Transmittance (550 nm) plotted as a function of sheet resistance (*R_s_*) for thin films prepared from four nanostructured materials, graphene, single-walled carbon-based nanomaterials, Ag nanowires (NWs) and Ag flakes. The dashed lines represent fitted plots of the bulk regime using Equation (5), while the solid lines corresponds to the percolative regime using Equation (6); (**c**) The date in Figure 2a are re-plotted on the axes of (*T*^−1/2^ − 1) and *R*_s_ on log-log scale. Note that (*T*^−^^1/2^ − 1) is proportional to film thickness. The dashed lines represent fitted plot of the bulk regime using Equation (5), while the solid lines corresponds to the percolative regime using Equation (6). (Reproduced with permission from [37]. Copyright American Chemical Society, 2010); (**d**) Haze factor (HF) *vs.* AF of Ag NWs with diameters of 56, 100, and 153 nm; (**e**) Slope of HF(%) *vs.* AF, HF(%)/AF, as a function of Ag NW diameter. (Reproduced with permission from [45]. Copyright AIP Publishing, 2013).

*T(%) =* 100 – *a×* AF (4)

where *T* is the transmittance of the film in %, the fitting parameter *a* accounts for the diameter and wavelength-dependent optical properties of the NWs, and AF is the area fraction of the NW films given by Equation (3). The fitted plots with Equation (4) are shown in Figure 2a with *a* = 87 and *a* = 61, which correspond to the diameters of 51 nm and 30 nm of Ag NWs, respectively [43]. The plots indicate that the transmittance of NW films increases with decreasing NW diameter and area coverage. As the area coverage of the NWs in a film approaches zero, the transmittance of the NW film approaches 100%.

In general, for NW conducting films, De *et al.* [37] proposed a simple model in which the transmittance is related to sheet resistance in the bulk and percolative regimes by the following equations:

(5) $$T={\left(1+\frac{1}{{П}_{bulk}}\frac{Z_{o}}{2R_{s}}\right)}^{-2}$$

(6) $$T={\left[1+\frac{1}{{П}_{percolative}}{\left(\frac{Z_{o}}{R_{s}}\right)}^{1/\left(n+1\right)}\right]}^{-2}$$

where *T* is the transmittance of the film, $Z_{o}$ is the impedance of free space, *n* is the percolation exponent, *R_s_* is the sheet resistance, ${П}_{bulk}$ and $\;{П}_{percolative}$ are the dimensionless number of the bulk and percolative regimes, respectively. Figure 2b shows *T* as a function of *R_s_* for thin films prepared from four nanostructured materials which are graphene, single-walled carbon nanotubes, Ag NWs, and Ag flakes. In most cases, the bulk-like behavior begins to occur at relatively low *R_s_* values which correspond to thick films (dashed lines) and does not occur in the technologically relevant regime (*T* > 90%), whereas the percolative regime can be identified as a straight line on a log-log plot of *(T*^−1/2^ *–* 1*)* *versus* *R_s_* (equivalent to a graph of thickness *vs*. *R_s_*) by Equation (6). The data for in Figure 2b are re-plotted with this form in Figure 2c. For each curve, the bulk and percolative regimes are clearly present, where *n* and Π can be obtained by fitting the data in both regimes. In short, high transmittance of NW films can be obtained with large Π values by decreasing the NW diameter [37].

In addition to transmittance as a critical optical property of TCFs, haze factor (HF) is becoming another important factor that affects the performance of TCFs. The haze factor (HF) is defined as [44]:

(7) $$HF=\frac{I_{s}}{I_{s}+I_{d}}\times 100(\%)$$

where *I_d_* is the light flux transmitted directly and *I_s_* is the flux of scattered light. The control of HF is an ongoing challenge for NW-based transparent electronic devices because NWs are strong scattering centers. Figure 2d,e show the HF values of NW films as a function of AF and the diameter of the NW, respectively. HF appears to scale linearly with AF, and it increases significantly with increasing NW diameter [45,46]. Furthermore, the haze value decreases almost linearly with increasing total transmittance [29]. Khanarian *et al.* proved that less than 1% HF of the Ag NW mesh film can be achieved if the diameter of the NW is less than 50 nm and the length is greater than 5 μm [45]. On the other hand, TCFs with different haze factors can be applied to different fields. For instance, large HF values are preferred for thin-film photovoltaic applications because an increase in the amount of light scattered from the electrode can enhance the coupling of light into the absorber material, thereby increasing the efficiency of the device [47], whereas in display applications, HF has to be kept to a minimum (usually <1%) to improve the clarity of a film [48].

Consequently, the optical properties of NW film are strongly dependent on the diameter and area coverage of the NWs on it. The relationship offers guidelines for developing a method of synthesizing NWs for optimal performance.

## 3. Synthesis of Cu NWs and Fabrication of Cu NW Thin Films

### 3.1. Synthesis of Cu NWs

Several methods for the synthesis of Cu NWs, such as chemical vapor deposition [49], vacuum thermal decomposition [50], and electro-spinning [51] have been reported. For the past ten years, chemical-solution methods (CSMs), including hydrothermal synthesis [52], reduction of a precursor solution [53], and catalytic synthesis [54] have emerged as important and mainstream routes to NW synthesis. CSMs have several advantages, such as fewer constraints regarding precursor selection and the conditions of the solvents and reactions, and the feasibility of large-scale production. Cu NW synthesis by CSMs at low temperature under ambient conditions is easier in terms of scaling up and commercialization than that by vacuum-vapor processes. All the CSMs are similar in that they use a reducing agent to transform metal ions into metal atoms and a capping agent to bring metal atoms into the NWs. In this section, we summarize the synthesis of Cu NWs by CSMs for use in TCFs. There are two general approaches: (i) the amine-assisted approach and (ii) the catalyst-assisted approach.

#### 3.1.1. Amine-Assisted Synthesis

**Figure 3:**
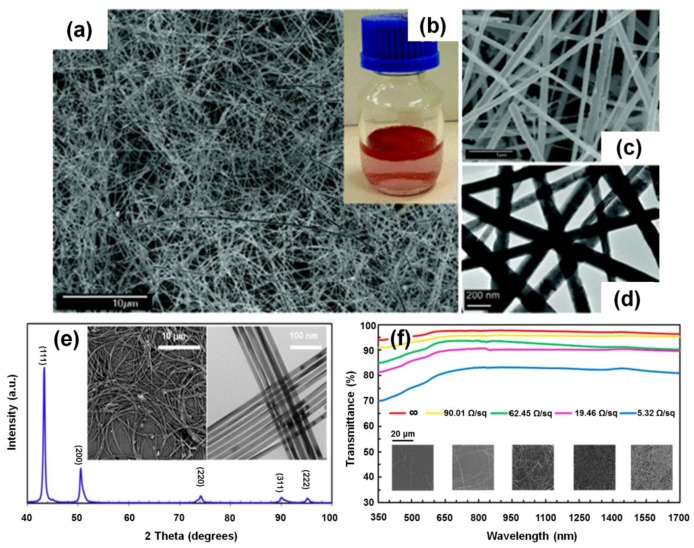
(**a**) Scanning electron microscopy (SEM) image of general views of Cu NWs; (**b**) Photographic image of as-prepared Cu NWs in mother liquor; (**c**) SEM image of detailed views of Cu NWs; (**d**) Transmission electron microscopy (TEM) image of Cu NWs (Reproduced with permission from [55]. Copyright American Chemical Society, 2005); (**e**) X-ray diffraction pattern of Cu NWs. Inset, SEM (**left**) and TEM (**right**) images of as-grown Cu NWs; (**f**) Wavelength-dependent transmittance, sheet resistance, and corresponding SEM images of transparent conductors. Substrate contribution is excluded. (Reproduced with permission from [29]. Copyright American Chemical Society, 2015).

Chang *et al.* developed one of the first solution-phase synthesis methods of Cu NWs [55]. The scanning electron microscopy (SEM) image, photographic image of mother liquor, magnified SEM and transmission electron microscopy (TEM) image of Cu NWs synthesized by their method are displayed in Figure 3a–d, respectively. The formation of Cu NWs in that work was based on the following redox reaction:

(8) $$Cu^{2+}\;+\;N_{2}H_{4}\overset{OH^{-},\;Heating,\text{ EDA}}{\to }Cu\;NWs$$

Cu^2+^ ions were reduced by hydrazine (N_2_H_4_) with ethylenediamine (EDA, C_2_H_8_N_2_) as a capping agent when a reactor containing highly basic copper nitrate (Cu(NO_3_)_2_) aqueous precursors was controlled at 25–100 °C. The resulting Cu NWs were straight, with uniform diameters in the range of 60–160 nm and lengths greater than 40 μm (Figure 3a–d). Rathmell *et al.* reported modification of this approach, focusing on large-scale synthesis produced Cu NWs 90 ± 10 nm in diameter and 10 ± 3 μm in length [8]. They also verified that the concentration of EDA is a critical parameter for anisotropic growth of NWs. When EDA was not added to the reaction, only Cu nanoparticles were produced. Shi *et al.* used copper(II) chloride (CuCl_2_) aqueous solution as a Cu^2+^ ion supplier, and employed octadecylamine (ODA, C_18_H_39_N) substituting for hydrazine and EDA as both a reductant and a surfactant [56]. The resulting Cu NWs had uniform diameters of 50–100 nm and lengths up to several millimeters. Jin *et al.* reported a procedure to synthesize Cu NWs by reducing Cu^2+^ ions with glucose in water with hexadecylamine (HAD) serving as a capping agent. [57]. They produced Cu NWs with controlled shapes, high purity, and good uniformity in relatively large quantities. The resulting Cu NWs had a uniform diameter of 25 nm and lengths ranging from several tens to hundreds of micrometers; some of them were as long as several millimeters. More recently, Cui *et al.* used oleylamine (OLA, C_18_H_37_N) both as a coordinating ligand for the CuCl_2_ precursor and a capping ligand on the Cu NW surface while tris(trimethylsilyl)silane (C_9_H_28_Si_4_) was introduced as a mild reducing reagent. Their method could produce ultrathin and highly crystalline Cu NWs with an average diameter of 17.5 nm and a length of 17 μm, which resulted in transparent Cu NW TCFs as shown in Figure 3e,f [29]. Another advantage of their approach is that the resulting Cu NW TCFs show a dramatically reduced HF due to the small NW diameter.

#### 3.1.2. Catalyst-Assisted Synthesis

The formation of Cu NWs using a catalyst-assisted synthesis is based on the following redox reaction:

(9) $$Cu^{2+}\overset{Catalyst\;\left(Ni,\;Pt\right),\;Amines,\;Heating}{\to }Cu\;NWs$$

These synthesis routes also employ amines, but differ from above-mentioned methods in that catalytic reactions occur during synthesis. Guo *et al.* successfully synthesized long and fine Cu NWs in OLA solution by the nickel(II) acetylacetonate (Ni(acac)_2_) catalytic procedure [58]. The resulting Cu NWs had high aspect ratios with diameters of 16.2 ± 2 nm and lengths up to 40 μm. Firstly, under heating at 175 °C, Ni^2+^ ions in the OLA solution were reduced to Ni atoms. Secondly, Cu atoms were formed by the galvanic replacement reaction of Cu^2+^ ions with Ni atoms; in this reaction, Ni^2+^ ions acted as catalysts. Lastly, Cu NWs grew slowly from Cu nanocrystal seeds and elongated in the presence of Cl^−^ ions from the CuCl_2_ precursor. The presence of Cl^−^ ions was indispensable because the Cl^−^ ions attached onto the (100) facets, resulting in strongly anisotropic growth. Zhang *et al.* synthesized ultralong crystalline Cu NWs with excellent dispersibility. through the self-catalytic growth of Cu NWs within a liquid crystalline medium of hexadecylamine (HAD, C_16_H_35_N) and cetyltrimethylammonium bromide (CTAB, C_19_H_42_BrN) [59]. The average diameter of Cu NWs produced by this method was 78 nm and the lengths varied from tens to hundreds of micrometers. It is noted that platinum (Pt) catalyst was indispensable for their method since no particles or NWs were observed without the catalyst even after long reaction time, which is attributed to the fact that the presence of Pt surface first catalyzes the reduction of Cu ions into metallic clusters or particles, which then migrate to the solution and act as seeds for growth of the NWs.

### 3.2. Fabrication of Cu NW Thin Films

The synthesized Cu NWs should be deposited on a substrate to fabricate TCFs. Vacuum filtration [31,58], spin coating [60,61,62], Meyer rod coating [30], nitrocellulose-based ink printing [63,64], drop-spray coating [65,66], and roll-to-roll printing [67] are the examples of general NW deposition processes regardless of the types of materials. However, the *R*_s_ value of the as-transferred or as-coated TCFs is generally high due to contact resistance between NWs. Several ways to minimize contact resistance to achieve lower *R*_s_ values of NW networks have been proposed.

**Figure 4:**
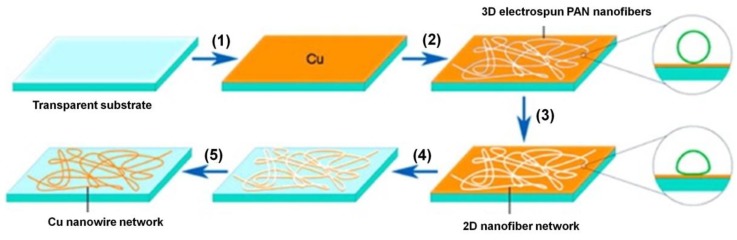
Scheme of Cu NW network electrode fabrication: (**1**) Electron-beam deposition of Cu film on the transparent substrate; (**2**) Electrospun polyacrylonitrile (PAN) nanofibers on the Cu-covered substrate; (**3**) Solvent vapor annealing. The insets show schematics of the fiber cross-sections before and after solvent vapor annealing; (**4**) Metal etching; (**5**) Removal of PAN fibers by dissolution. Cu NW network on transparent substrate after PAN is removed (Reproduced with permission from [73]. Copyright American Chemical Society, 2014).

**Figure 5:**
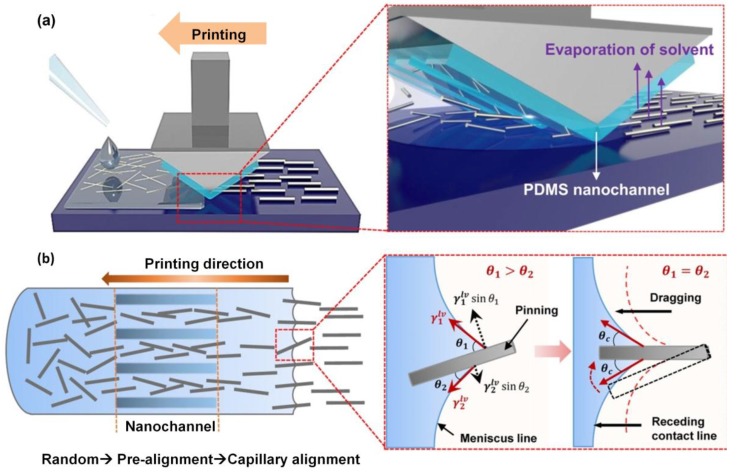
Solution-printed highly aligned Ag nanowire (NW) arrays: (**a**) Schematic of the capillary printing process using a nanopatterned polydimethylsiloxane (PDMS) stamp to produce highly aligned NW arrays; (**b**) Schematic showing the alignment process during capillary printing of unidirectional NW arrays. The solvent-evaporation-induced capillary force produces highly aligned networks by dragging confined NWs at the solid-liquid-vapor contact line. (Reproduced with permission from [68]. Copyright American Chemical Society, 2015).

He *et al.* reported a new approach by which tough and excellent-performance Cu NW transparent electrodes were fabricated using electrospun fibers as a mask [60]. As shown in Figure 4, electrospun nanofiber networks provide percolating paths that result in completely connected metal NW patterns, which in turn lead to high-conductivity networks. Interestingly, the performance of the transparent Cu NW electrodes in their work is better than that of ITO, CNTs, and graphene-based electrodes in that the Cu NW films showed excellent adhesion to the substrate, offering the benefit of being usable on polymer substrates. In addition, the low fabrication cost and simplicity of electrospinning make it a promising method to produce large-scale transparent electrodes. Han *et al.* introduced ultrafast plasmonic nanoscale welding by using a polarized laser under ambient conditions. A Cu NW percolation network was first formed on various substrates from a Cu NW solution, followed by laser irradiation, which generated laser-induced heat at the junctions of each NW due to the plasmonic effect. As a consequence, the resistance of Cu NW network can be reduced significantly because the NW junctions were welded together [31]. The electrical performance of NW TCFs can also be improved by NW alignment. Kang *et al.* [68] introduced a capillary printing technique for the fabrication of transparent electrodes using Ag NWs (Figure 5). The Ag NW solution was deposited and dragged to generate uniformly aligned NWs by controlling the nanochannels. The aligned NWs were predicted to have a significantly lower percolation threshold, which would improve the transmittance and conductivity of the film. Although their process was applied to Ag NW alignment, the same process can possibly be used for Cu NWs. In addition, mechanical pressing [69,70], thermal annealing [71], and optothermal heating [72], reported to improve electrical performance of Ag NW electrodes, can be applied to Cu NW electrodes as well.

The roughness of a NW film is another important factor for device applications because high roughness of a TCF surface leads to leakage or short circuits [71]. NW films have a surface roughness of at least two NW diameters because NWs stack in junctions after they are transferred from the solution to the substrate [74]. Researchers have found various ways to address this issue, such as polymer coating [75], thermal annealing [70], and mechanical pressing [63]. These approaches can generally be applied regardless of the types of materials.

Adhesion of NWs to a substrate also affects device performance because NWs with poor adhesion can be wiped off the substrate, resulting in malfunction of the device. The degree of adhesion depends on various factors such as the chemical and physical properties of the NW, the type of the substrate, and post-processing. Poor adhesion can be solved by embedding the NWs into the surface of the polymer matrix [74,76], or by encapsulating NWs within a thin layer of organic materials [65]. Because the electrical properties of NWs are also affected by the embedment and encapsulation, proper treatment should be carefully applied.

## 4. Protection of Cu NWs Films against Oxidation

**Figure 6:**
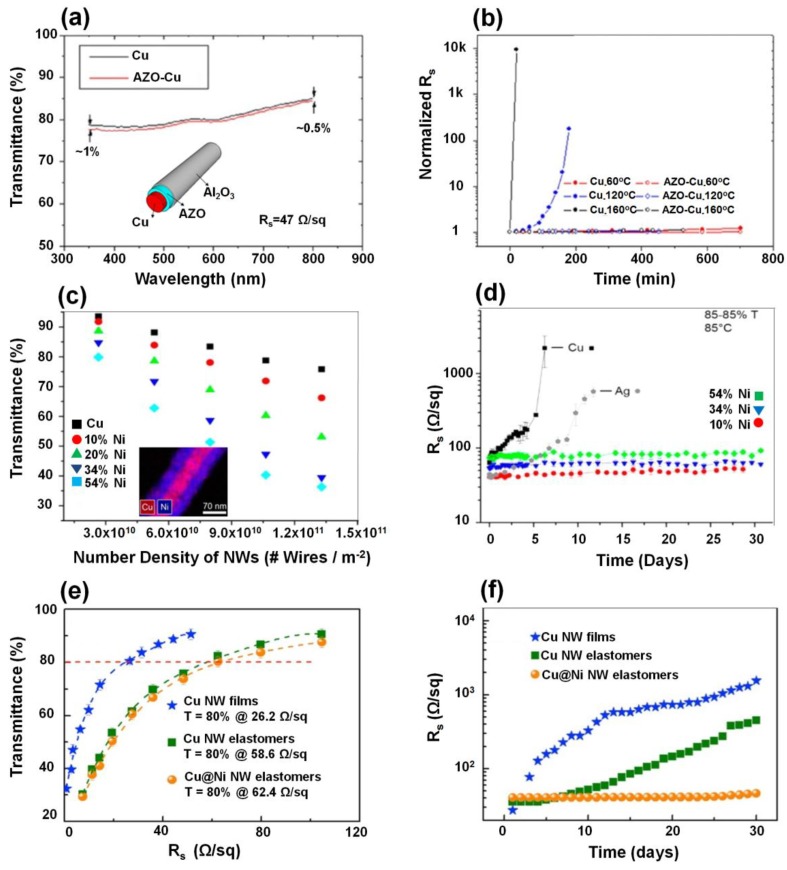
(**a**) Transmittance of electrospun Cu nanofibers before and after atomic layer deposition (ALD); inset is a cartoon (not to scale) of the Cu@AZO/Al_2_O_3_ nanofiber (**b**) Normalized sheet resistance (*R*_s_) of bare and protected Cu NWs *vs.* baking time at 80, 120, and 160 °C. Solid circles refer to bare Cu nanofibers and hollow circles represent AZO-Cu nanofibers. In the case of bare Cu nanofibers at 120 and 160 °C, the resistance exceeded the multimeter limit (20 MΩ) in the end. (Reproduced with permission from [77]. Copyright American Chemical Society, 2012); (**c**) A plot of specular transmittance *vs.* number density of NWs, which shows the effect of increasing wire diameter on the film transmittance; inset is an energy-dispersive X-ray spectroscopy (EDS) mapping image of a cupronickel NW coated with 54 mol % nickel; (**d**) Plot of *R*_s_ *vs.* time for films of Ag NWs, Cu NWs, and cupronickel NWs stored at 85 °C (Reproduced with permission from [78]. Copyright American Chemical Society, 2012); (**e**) Transmittance as a function of *R*_s_ for various NW electrodes; (**f**) *R*_s_ *vs.* time for various NW electrodes during exposure in the natural environment. (Reproduced with permission from [81]. Copyright American Chemical Society, 2014).

The most critical issue of Cu NWs is that Cu easily reacts with oxygen under ambient conditions; it thus oxidizes and becomes non-conductive. Furthermore, as the temperature increases, the oxidation rate accelerates. For this reason, protection of Cu NW films from atmospheric corrosion is the most crucial challenge for researchers in their attempts to apply Cu NWs to commercial production. Hsu *et al.* tried to solve this issue by using atomic layer deposition (ALD) to coat a passivation layer of aluminum-doped zinc oxide (AZO) and aluminum oxide (Al_2_O_3_) onto electrospun Cu NWs, as shown in the inset of Figure 6a [77]. In their work, the AZO layer improved the reliability of Cu NWs owing to its high transparency and conductivity, whereas the outer ultrathin Al_2_O_3_ layer enhanced the chemical resistance of Cu NWs to corrosion and oxidation. The core-shell structure of Cu_core_-AZO/Al_2_O_3-shell_ NWs retained the same resistance before and after ALD, and the transmittance decreased by only 1% (Figure 6a). This passivation layer therefore had a remarkable effect in protecting Cu NWs from thermal oxidation at high temperatures up to 160 °C in dry air and up to 80 °C in humid air (Figure 6b). Rathmell *et al.* reported solution-phase synthesis of Cu_core_–Ni_shell_ NWs with several Cu to Ni ratios [78]. The transmittance of the Cu NWs coated with Ni in a Cu:Ni ratio of 1:1 decreased from 94% to 84% (Figure 6c) at the low *R*_s_ of 60 Ω/sq. However, the Cu_core_–Ni_shell_ NW films show 1,000 times more resistance to oxidation than the Cu NW films. As shown in Figure 6d, without any Ni coating, the *R*_s_ value of the bare Cu NW-based TCFs began to increase after 1 day in a dry oven at 85 °C. The *R*_s_ value soared by an order of magnitude after 5 days, whereas the *R*_s_ value of the Ag NW films increased by an order of magnitude after 13 days. In comparison, the *R*_s_ value of the Ni-coated Cu NW-based TCFs remained remarkably stable over a period of 30 days. On the other hand, Chen *et al.* coated Zn, Sn, and In onto Cu NWs by a solution-phase process in order to protect Cu NW films from oxidation without degrading the optoelectric properties of the film [79]. Coating Cu NWs with a complete shell of Zn, Sn, or In usually leads to a decrease in the transmittance of the film of 4%–9%; however, this transmittance loss can be largely recovered by exposing the core-shell NWs to oxidizing conditions, or H_2_O_2_ treatment. For instance, treatment of a Cu_core_–Zn_shell_ NW film in a 2 wt % solution of H_2_O_2_ in H_2_O for 15 min increased the transmittance of the film from 76% to 84% while maintaining the low *R*_s_ value of the film. Mehta *et al.* developed a novel method using low-temperature plasma-enhanced chemical vapor deposition (CVD) to coat uniform graphene onto the surfaces of Cu NWs [80]. Graphene has remarkable in-plane stiffness to prevent mechanical deformation and impermeability to protect Cu against chemical reactions. A strong enhancement of both electrical and thermal conductivity in graphene-encapsulated Cu NWs was observed because passivation of the Cu surface with graphene induced partial elastic surface scattering, which consequently resulted in an increase of electron transport through the NWs. Recently, Song *et al.* demonstrated conductive Cu@Cu–Ni NW elastomer composites with ultrahigh performance stability against oxidation, bending, and stretching. The Cu@Cu–Ni NWs were fabricated by one-pot heating of CuCl_2_ in an OLA solution of Ni(acac)_2_ to achieve high performance with a transparency of 80% and an *R*_s_ value of 62.4 Ω/sq (Figure 6e). During the 30-day measurement, the *R*_s_ values of the Cu@Cu–Ni NW composites remained at 20 Ω/sq, and the fluctuation amplitude was within 2 Ω/sq (Figure 6f). Furthermore, the lifetime of the Cu@Cu–Ni NW elastomer composites was estimated to exceed 1200 days, which was one of the longest stable durations of Cu NW-based electrodes [81].

More recently, it was revealed that laser irradiation [82] can turn Cu*_x_*O NWs back into Cu NWs. Those processes can be performed rapidly at room temperature without using any vacuum process. The resulting Cu NWs exhibit superior conductivity with better resistance to the environment due to the enhanced Cu NW junctions. As a result, Cu*_x_*O NWs can be employed as starting materials for Cu NW thin films, thereby facilitating long-term storage and versatile applications of the material.

## 5. Applications and Perspective

As investigated above, Cu NW-based TCFs have shown excellent performance in terms of optoelectronic properties and mechanical flexibility. Therefore, they have potential application in various optoelectronic devices such as touchscreens [31,83], solar cells [63,84,85,86], OLEDs [81,87,88], and sensors [89,90]. Stewart *et al.* showed that organic solar cells (OSCs) using Cu_core_–Ni_shell_ NW films as transparent anodes can achieve a device efficiency of 4.9%, nearly equivalent to the 5% efficiency of OSCs utilizing Ag NW anodes [63]. Won *et al.* also successfully adopted Cu NW-based transparent electrodes into Cu(In_1−*x*_,Ga*_x_*)(S,Se)_2_ thin-film solar cells, which exhibited a power conversion efficiency of 7.1% [85]. Song *et al.* demonstrated usage of Cu@Cu–Ni NWs as conductors in OLED devices that were stable under extreme bending and stretching conditions [81]. For sensor applications, Cu NWs have been widely investigated in the development of electrochemical biosensors due to their efficient electron transfer and good mechanical strength. Stortini *et al.* prepared an ensemble of Cu NW electrodes with highly controlled morphologies. The electrodes demonstrated efficient sensing performance with respect to nitrate determination at concentration levels as low as a few micromoles [89]. As for touchscreen applications, Han *et al.* reported a resistive-type touchscreen device employing Cu NWs as a transparent conductor [31].

The required optical and electrical properties of TCFs can be different for each application. For example, touchscreens often require an *R*_s_ value of 50–100 Ω/sq and a transmittance of more than 85% along with a low HF, whereas solar cell applications require an *R*_s_ value below 20 Ω/sq and a transmittance of more than 90% [91].

**Figure 7:**
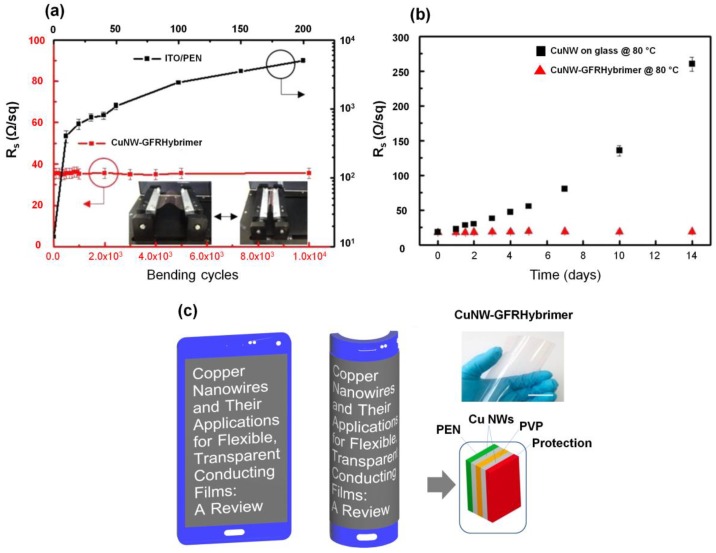
(**a**) Bending test results of a GFRHybrimer film (sheet resistance, *R*_s_ = 35 Ω/sq) and a reference indium tin oxide (ITO)/polyethylene naphthalate (PEN) film (*R*_s_ = 15 Ω/sq). Top and right axes are for the ITO/PEN (the inset represents the experimental setup for the bending test; bending radius is 5 mm); (**b**) Thermal and oxidation stability tests of bare Cu NW film and CuNW-GFRHybrimer film at 80 °C; (**c**) A schematic example of flexible smartphone using Cu NWs in the future. The inset is a photograph of a Cu NW-GFRHybrimer film (scale bar is 3 cm). (Reproduced with permission from [88]. Copyright American Chemical Society, 2014).

The excellent mechanical durability of Cu NWs embedded on the surface of a transparent glass-fabric reinforced plastic (GFRHybrimer) was confirmed by cyclic bending (Figure 7a). The long-term thermal and oxidation stability test of CuNW-GFRHybrimer revealed no drastic change in electrical performance after about 15 days (Figure 7b). Accordingly, we can expect to meet the demand of daily-use portable devices by adopting flexible Cu NW touchscreen panels in the near future as illustrated in Figure 7c.

ITO has been the most widely used material for TCFs for a long time, and it will continue to play a vital role in the future. However, Cu NW films that are durable and flexible and that enhance the optoelectronic properties with lower production cost are also a possibility. In this regard, Cu NWs stand as a strong alternative to ITO; therefore, in the future, there will be immense opportunities for exploring new designs using Cu NW-based TCFs, including flexible displays and curved surfaces.

## 6. Conclusions

Cu NWs have emerged as the promising next-generation conducting materials due to their excellent performance with high conductivity, high transmittance, mechanical flexibility, and cost-effectiveness. In terms of transmittance and *R*_s_, Cu NW films are already comparable to commercial ITO films while also exhibiting much better flexibility that allows devices based on Cu NWs to be bent or stretched. These superior characteristics make Cu NWs strong competitors for use in flexible electronics in the future. In this review, we briefly summarized the relationship between the dimensions and optoelectronic properties of NWs, and examined the control of Cu NW structure by various synthesis and fabrication routes for optimal performance of Cu NW films. We also discussed the development of new approaches to eliminating the wire-wire junction resistance between Cu NWs for better electrical performance. There are still challenges that need to be overcome, such as oxidation, haze, and large scalable fabrication, before Cu NWs can be fully integrated into commercial devices. In the future, further studies that cover the following aspects would be required: (a) the long-term stability of Cu NWs against oxygen, high temperatures, chemical etching, and mechanical strains; and (b) the fine-tuning of the structure and facile synthesis of Cu NWs. Once these problems are solved, Cu NWs as the foundation of next-generation TCFs can be expected.
